# Implementation of Pharmaceutical Technical Assistants on Hospital Wards and Their Impact on Patient Safety and Quality of Care: A Qualitative Study on Nurses' Experiences and Perceptions

**DOI:** 10.1155/2024/7894331

**Published:** 2024-06-12

**Authors:** Marjan De Graef, Brecht Serraes, Veronique Van Rompay, Nienke E. Dijkstra, Eibert R. Heerdink, Tinne Dilles

**Affiliations:** ^1^Clinical Nursing and Allied Health Research and Development Group (CNuAH-RD), Nursing and Paramedical Department, Vitaz Hospital and Health Care, Moerlandstraat 1, Sint-Niklaas 9100, Belgium; ^2^NuPhaC, Nurse and Pharmaceutical Care Consortium, Universiteitsplein 1, Wilrijk 2610, Belgium; ^3^Department of Nursing Science and Midwifery, Centre for Research and Innovation in Care (CRIC), Faculty of Medicine and Health Sciences, University of Antwerp, Universiteitsplein 1, Wilrijk 2610, Antwerp, Belgium; ^4^Research Group Innovations in Healthcare Processes in Pharmacology, University of Applied Sciences Utrecht, P.O. Box 12011, 3501 AA, Utrecht, Netherlands; ^5^Division of Pharmacoepidemiology and Clinical Pharmacology, Utrecht Institute for Pharmaceutical Sciences, Utrecht University, P.O. Box 80082, 3508 TB, Utrecht, Netherlands

## Abstract

**Methods:**

In a qualitative descriptive study, between December 2022 and March 2023, 16 semistructured interviews were carried out with a stratified purposive sample of nurses across internal, surgical, and geriatric wards. The inclusion criteria required a minimum of six months of work experience and experience working both day and night shifts. Inductive thematic analysis was performed in NVivo 1.6.1.

**Results:**

Semistructured interviews revealed three main themes: (1) patient safety and quality of care, (2) organization of care, and (3) role development and collaboration. The implementation of pharmaceutical technical assistants on nursing wards was perceived to reduce the risk of medication errors without compromising care quality, allowing nurses to spend more time on direct patient care. Clear communication procedures were vital for successful implementation, highlighting the need for collaboration and information exchange between pharmaceutical technical assistants and nurses. Continuity in assigning pharmaceutical technical assistants was highlighted as crucial to improve medication safety and quality of care. This is considered an important aspect to ensure a smooth and optimal cooperation between nurses and pharmaceutical technical assistants. Nurses expressed that working with pharmaceutical technical assistants challenged their supervisory role and teamwork dynamics.

**Conclusions:**

Nurses confirmed the added value of pharmaceutical technical assistants in medication management. Critical factors included dedicated assignments to hospital wards, clear roles, and mutual expectations in collaboration with ward nurses.

## 1. Introduction

The medication process on hospital wards is a routine but complex activity, involving multiple steps (prescription, dispensation, preparation, double-checking, administration, and evaluation) and various healthcare professionals. However, a critical workforce crisis is growing, with a projected global shortage of 7.2 million healthcare professionals expected to increase to 12.9 million by 2030, including a shortfall of 5.9 million nurses [1, 2]. Nurses have a unique role in medication administration [3, 4]. In the medication process, there are specific actions for nurses (such as administering), but there is also overlap in actions that can be performed by other healthcare professionals (e.g., dispensing and preparing).

At the dispensing and administration stage, errors are common (32–38% of all medication errors that harm patients) [5]. Medication errors are defined as preventable events that (have the potential to) harm the patient [6]. Medication errors can be caused by structural factors in nursing practice, including high workload, stressful environments, distractions, multitasking, and interruptions [7–10]. Sheik et al. [11] found disruptions during patient care contribute to half of hospital medication errors in patient files.

Studies suggest that pharmaceutical technical assistants (PTA) can contribute to hospital wards to a substantial improvement of medication dispensation [12–15]. Beyond dispensing, PTAs may perform other roles in a hospital. A literature review suggests that through the use of PTAs within medication reconciliation, hospitals experienced cost savings and other healthcare professionals saved time for other patient care activities, as well as increased trust in the accuracy of medication history [16].

A general hospital in Belgium, Vitaz, implemented PTAs to achieve high-quality medication service and allow nurses, physicians, and pharmacists to practice at the highest level of their licence. Between January 2021 and June 2022, ten PTAs were assigned to six hospital wards from Monday through Friday. Each PTA had two hours of daily medication dispensing and making stock corrections for the coming 24 hours. Nurses remained responsible for medication control and administration to patients [17].

Implementing PTAs on hospital wards can improve medication dispensation. Studies reveal that nurses, involved in direct patient care, experience more interruptions than PTAs. This allows PTAs to focus on dispensing medication [15, 18]. The main reason for implementing PTAs on hospital wards is to improve the job characteristics of nurses by reducing their workload and releasing additional time for direct patient care [15]. The integration of PTAs changes the organizational structure of a hospital ward, which can contribute to avoiding medication errors. Furthermore, this integration may lead to job enrichment and job satisfaction of the hospital PTAs [19, 20].

The PTA in Belgium, recognized since 1997, is a paramedic professional who is responsible for the dispensation of medication under the supervision of a pharmacist. Education, with at least 300 hours of practical internship in a pharmacy, is scaled to level 4—technical secondary education in the European qualification framework and the International Standard Classification of Education [21, 22]. A Belgian PTA is certified to perform acts, such as receiving and registering prescriptions, medication delivery, informing patients about adequate and safe use, making preparations, and training PTAs [23].

To evaluate and improve the role and implementation of PTAs on the hospital ward, nurses are key stakeholders. Nurses experience changes in organizational structure on a daily basis, can detect potential risks, and suggest improvements. This study aimed to explore nurses' experiences and perceptions of the implementation of PTAs on hospital wards to support the medication dispensation process, role development, and impact on safety and quality of care to determine critical success factors and opportunities for quality improvement.

## 2. Methods

### 2.1. Study Design, Setting, and Period

A qualitative study with individual semistructured interviews took place on hospital wards (December 2022–March 2023). These wards had PTAs assigned during this study or between January 2021 and June 2022.

### 2.2. Participants and Sample Procedure

In this study, 16 semistructured interviews were carried out with a stratified purposive sample of nurses across internal, surgical, and geriatric wards. The inclusion criteria required a minimum of six months of work experience and experience working both day and night shifts. Participants were informed about the study through written and oral communication, including e-mail, telephone, and face-to-face interactions. Data saturation was assessed through consensus among the project group members (MDG, BS, TD, RH, and ND) and was achieved after conducting 16 interviews.

### 2.3. Data Collection

The interview guide was informed by a review of the literature on the roles of PTAs on hospital wards. Open-ended questions aimed for participants to freely express their personal experiences on organization, work environment, collaboration, role development, criteria, and potential benefits of the implementations of PTAs on hospital wards. The interview guide was discussed and refined at regular project group meetings. All interviews were conducted by MDG or VVR, audio recorded, pseudonymized, and transcribed verbatim. The interviews were held in a separate and quiet room. The accuracy of the transcripts was reviewed by proofreading by the two researchers. To describe the population, the following demographic data were collected: sex, age, education level, years of experience, and current job time.

### 2.4. Data Analysis

Inductive thematic analysis was conducted in NVivo 1.6.1 to uncover emerging patterns and (sub)themes from the data. Two researchers (MDG and VVR) independently coded the data to enhance the credibility of the findings. After analysing the first four interviews, the researchers held a cross-check meeting to ensure consistency in coding strategies and refine approaches, such as clustering codes and formulating (sub)themes. This iterative process continued throughout data collection, with a final meeting held when both researchers independently reached data saturation, indicating that no new themes were emerging. To mitigate potential biases in data gathering, analysis, and reporting, the preliminary analysis findings were presented to the project group for further discussion. Finally, to ensure a thorough exploration of the identified themes and subthemes, all project group members engaged in comprehensive discussions until consensus on the final thematic framework was achieved.

### 2.5. Credibility and Trustworthiness

Data triangulation involved a meticulous cross-validation process. To allow critical and sense-making discussion of data interpretations, regularcross-check meetings between the two researchers were held. In addition, discussions within the project group provided a valuable collaborative perspective that served to validate the findings and mitigate potential bias. After the analysis, the findings were presented to the participants using a thematic presentation of the themes. The participants discussed these findings to reduce the risk of superficial or false interpretation and confirmed that the quality of data analysis was rigorous and reliable. This interactive dialogue provided a deeper exploration of the participants' experiences and insights, reducing the risk of superficial or false interpretations, and ensured that the data analysis was embedded in the participants' experiences.

### 2.6. Ethical Considerations

The study adhered to the Declaration of Helsinki and received approval from the Ethics Committee of Vitaz (registration number EC/22054) [24]. The study was reported according to the Consolidated Criteria for Reporting Qualitative Studies (COREQ) checklist for qualitative research [25].

## 3. Results

Seven head nurses and nine nurses were interviewed for a total of 347 minutes, with a mean duration of 21.7 minutes (range 18–29) per interview. The majority of the interviewees were females (*n* = 11, 69%), with a mean age of 40.8 years, and 13 (81%) participants had more than five years of work experience (Table 1).

**Figure 1:**
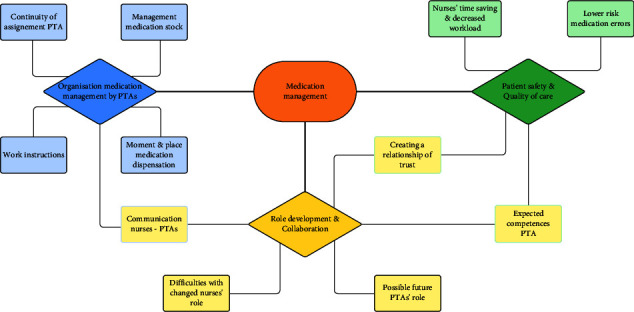
Main themes and interconnected subthemes of nurses' perception of the implementation of PTAs to support the medication management process on hospital wards.

Nurses had a positive perception of the implementation of PTAs to support the process of medication management on hospital wards. All participants endorsed the content, formulation of themes, and deeper insights in the thematic presentation and discussion of the themes. Substantial changes were not required after this presentation. The analysis identified three main themes: (1) organization of medication management by PTAs, (2) patient safety and quality of care, and (3) role development and collaboration (Figure 1). These main themes are divided into several subthemes and described hereafter.

### 3.1. Organization of Medication Management by PTAs

In terms of the organization of medication management by PTAs on the hospital ward, five subthemes were described by nurses' experiences: (1) work instructions, (2) moment and place medication preparation, (3) continuity of the assignment of PTAs, (4) management medication stock, and (5) communication.

#### 3.1.1. Work Instructions

Nurses mentioned misunderstandings of PTAs' responsibilities due to unclear insight of PTAs' time on the ward. Nurses emphasized the need for clear and well-defined work instructions for optimal medication dispensation. These work instructions are fundamental for all healthcare professionals to understand and set expectations for PTAs on the hospital ward.

Nurse 5: “Sometimes there are misunderstandings. Like the other day, our PTA said they are not allowed to take from the home medication to prepare. But those are things we as nurses don't know, unless we verify it with our pharmacist.”

Nurse 13: “They actually prepare all the medication according to the medication program. I don't think there are really concrete agreements.”

#### 3.1.2. Moment and Place Medication Dispensation

The timing and location of the medication dispensation by PTAs emerged as a notable consideration in the organization of the medication dispensation on the hospital ward. At the initial start of the implementation of PTAs, they performed their tasks on the hospital ward in the afternoon. Later, to be able to provide more hospital wards with this pharmacy service, the organization made operational adjustments by sending PTAs to some hospital wards in the morning.

Nurse 3: “It's always busy in the same rooms. That place is too small and everything comes together. The telemetries go off constantly, nurses come there to wash their hands, the shift change, etc. There should be walls, a really separate room, that would make a difference. In fact, that's how it should be to prepare medication in completely enclosed rooms.”

Participants indicated that the moment of medication dispensation by the PTAs influences the workload of nurses and their ability to provide direct patient care. When PTAs dispense the medication for the following day in the morning, there is a higher risk of medication changes than when this is done in the afternoon, especially after the doctor's round, when medication use is evaluated. Nurses mentioned that greater awareness of the moment of medication dispensation by PTAs is critical to prevent night nurses from spending, again, additional time supplementing and reviewing the medication dispensed by PTAs. Careful consideration of the optimal time for medication preparation by PTAs is crucial to maintain quality of care, minimize interruptions, and reduce the risk of medication discrepancies.

Nurse 15: “At this point they come to prepare in the morning, if an emergency admission occurred the day before, you have not received medication yet, because it does not arrive until the afternoon. If the doctor prescribes something in the morning, they have not seen it, because they have been there before the doctor has made all the adjustments… Things are really not going well on the ward at the moment.”

To achieve high-quality medication dispensation, participants mentioned the importance of an enclosed, quiet medication room on the hospital ward. These environmental conditions were seen as essential elements to concentrate and perform their tasks with precision. The quiet condition not only allowed the PTAs to focus on their task but also served as a protective barrier against distractions or interruptions, which can potentially contribute to medication errors.

#### 3.1.3. Continuity of Assignment PTAs

The continuity of assignment in terms of daily allocation of PTAs and in terms of the medication management process recurred frequently throughout the interviews. PTAs can be allocated to different wards every day; however, all participants perceived that the structural allocation of PTAs to the same hospital wards could have many advantages. The potential benefit of this approach is a closer and more collaborative relationship, effectively forming a unified team that aims to build trust between nurses and assigned PTAs. In addition, nurses highlighted that when PTAs are consistently assigned to the same hospital wards, they have the opportunity to improve their clinical reasoning skills, which may contribute to the quality of care delivered.

Nurse 3: “The biggest frustration of nurses: we are here 24/7 and every discipline that comes to support us is limited.”

Different opinions arise on the availability of PTAs during the week or weekends. Some express frustration over the fact that PTAs are only available on weekdays when nursing care is a requirement 24/7. On the contrary, other participants are of the opinion that PTA support might not be as essential during weekends and holidays, noting a perceived decrease in workload. Participants indicated that these varying perspectives are often linked to the unique needs of individual hospital wards. Surgical wards, for example, tend to experience a higher patient turnover during the week compared to weekends and holidays, leading to a lower workload on weekends and holidays. Geriatric wards often experience continuous bed occupancy, maintaining a consistent workload throughout the week, unaffected by specific days.

Nurse 5: “Especially during the week, because on the weekends you have fewer patients, normally, and on the other hand, speaking for myself, you are pulling the strings yourself again.”

Nurse 11: “It would be easier if you have someone specific, or a couple of pharmacy assistants, who alternate specific on your hospital ward. Because you don't see someone else every day, but you can build a relationship of trust.”

#### 3.1.4. Management Medication Stock

Most nurses agreed that the introduction of PTAs improved the accuracy and efficiency of medication stock management on the hospital ward. PTAs overseeing this aspect reduced nursing time spent on frequent pharmacy calls for dispensing, restocking, or addressing missing medications. However, it is important to note that not all nurses shared the same perspective. Some nurses believed that PTAs could improve their role by ensuring that the necessary medications for the next 24 hours are in stock on the ward. This perspective emphasizes the ongoing pursuit to refine the balance between medication stock management and anticipating the specific needs of the ward.

Nurse 3: “Make sure that your antibiotics or important medications are in stock for the first 48 hours, so that the next shift does not have to call ‘we're on our last medication'.”

Nurse 16: “If they see that we have shortages in the closet, they pass it on to their colleagues and then that medication comes faster to the hospital ward. I have to call a lot less to the pharmacy to tell them there is a shortage of medication stock. That's something I really notice.”

#### 3.1.5. Communication between Nurses and PTAs

The subtheme “communication” not only plays a vital role in organizing medication management, but also has far-reaching implications for role development and collaboration. All participants mentioned that effective communication between nurses and PTAs is essential to ensure patient safety and effective implementation.

To improve adequate communication between PTAs and nursing staff and mitigate the risk of medication errors, the hospital introduced a communication tool called “medication-attention cards.” These cards were designed to be placed within patient medication trays, highlighting crucial information, such as missing doses (ordered by hospital pharmacy), multidose medications, or medications on a medication strip. However, most of the participants expressed doubts about the effectiveness of this approach. There was often uncertainty about interpreting these medication-attention cards, and their inclusion was seen to potentially clutter and confuse the medication tray. On the contrary, nurses noted the critical need for a simplified and efficient method to facilitate the exchange of medication information between PTAs and nursing staff.

Nurse 2: “There are often little cards inside, pink, blue, and yellow… That means something like multidose medication, like insulin. But we don't really know exactly what those cards mean, what medication they are about.”

Nurse 15: “That pink one, that is for the ordered medication. If the pharmacy has already seen it but does not have it, they place that to draw attention to the night nurse, ‘here should be an additional pill'.”

### 3.2. Role Development and Collaboration

This theme is divided into five subthemes: difficulties with changed nurses' role, team, communication, competencies of PTAs, and possible future PTAs' role.

#### 3.2.1. Difficulties with Changed Nurse's Role

The implementation of PTAs into the medication dispensation process represents a substantial task shift and introduces role differentiation between PTAs and nurses. This transition led to a decrease in overall workload; an improvement acknowledged by all participants. However, an interesting and nuanced perspective emerged among some participants who found it challenging to completely disengage from medication dispensation responsibilities, particularly during the night shift. These participants mentioned a sense of responsibility to ensure the accuracy of medication dispensation for their colleagues who worked the following day. This feeling of accountability extended to maintaining a clear overview of their patients' medication regimens, recognizing their critical role in intercepting potential errors in medication prescriptions, especially concerning oral and subcutaneous anticoagulant, double antibiotics, etc. In addressing these concerns, many participants indicated a practice they adopted during the night shift: a “quick check” of medications dispensed by PTAs. This creates an additional third check before the medication reaches the patient. Although this is an additional and unattended step, this improvised verification process was perceived to provide an additional layer of medication safety.

Nurse 12: “Like today, the morning shift has already prepared half of the medication and the evening shift is going to complete the other half, so the night shift can be reassured: they only have to perform a last check and prepare the medication for new admissions.”

Exactly for that reason, several participants mentioned their willingness and even satisfaction with the responsibility of dispensing medication on weekend and holiday nights. This allowed them to periodically review and maintain a clear medication overview, minimizing the potential risk of losing track of medication details. In essence, it provided a resource for nurses to address proactively any discrepancies or potential errors in medication prescriptions, reaffirming their commitment to patient safety. Interestingly, there are also certain hospital wards where day nurses now undertake the task of medication dispensation for the following day when there are no PTAs available to support them.

Nurse 15: “We are much less aware of the medications the patients are taking. As a night nurse you sometimes see things like ‘he is taking that and that together' or ‘he complains about that, but he gets this and this'. we miss that a bit by not preparing it anymore. Of course, at busy times we are happy that it is already done, but sometimes we miss not having a good overview of patients' medication. Some things are important to keep in mind.”

#### 3.2.2. Creating a Relationship of Trust

The participants mentioned the importance of assigning PTAs to the same hospital wards as an important factor for closer collaboration and integration into the healthcare team. The participants perceived that when the same PTAs worked on their hospital ward, they could learn to know each other and begin building a relationship of trust. However, all participants indicate that there is pleasant contact with the PTAs, and they do not have the feeling to be one team. Although PTAs have a uniform way of working on all the different hospital wards, participants appreciate it when PTAs make small adjustments in their working methods to accommodate the habits of the ward. For example, a hospital ward has the habit of using medication jars with different colours for each moment of the day. On this ward, PTAs and nurses agreed to prepare the medication for the next day in coloured jars, provided that the nurses already had the jars ready to go.

Nurse 1: “They should be part of your team. I know we will not be the only team. I think they will probably work on two or three hospital wards. It is a matter of creating that bond of trust and that includes communication. That is just the way it is, and that trust needs to be created. That is something that needs to grow, you cannot force it.”

Nurse 14: “Unfortunately, it doesn't seem like that, it seems a bit separate. But actually, I think that, if you work with permanent people, they will really become a part of the team.”

#### 3.2.3. Communication between Nurses and PTAs

All participants perceived that effective communication among various healthcare professionals is the key to successful working collaboration. They recognized four important aspects of communication: content, participants, time, and moment. The presence of a clear and standardized procedure for each of these aspects is important for all participants. Most of the participants expressed their desire for regular consultation moments, which they perceived as opportunities to exchange feedback in both ways. They recognized the importance of these moments in facilitating an open and constructive dialogue, enabling each professional to share insights, perspectives, and concerns.

Nurse 3: “Maybe ask for feedback: ‘was everything clear?', ‘I prepared the medication yesterday, do you have comments for me?' People rarely ask for feedback themselves; it can work well on both sides, I think.”

#### 3.2.4. Expected Competencies of PTAs

Comprehensive awareness of the competencies and authorizations of the PTAs is lacking among nurses. They described that this lack of clarity has contributed to their hesitancy to seek guidance from PTAs about the medication of their patients. Nurses shared a collective expectation for more clinical reasoning skills among PTAs and seeing them as integral members of the healthcare team. Therefore, participants emphasized the need for clarity on the core competencies and cognitive processes expected from PTAs. This clarity, they believed, should be integrated into supplementary education, assessment, and practical training for PTAs.

Nurse 2: “I don't actually know what kind of training they have had. What do they know? Can we ask them questions about medication or do we really have to call the pharmacy for that?”

Nurse 3: “Basic training, clinical reasoning. A crash course: always look at the context of your patient, if in doubt, consult with the nurse who probably knows the patient better.”

#### 3.2.5. Possible Future Roles of PTAs

The lack of comprehensive knowledge on the competencies and authorizations of the PTAs was also reflected when participants were asked about the potential to expand the roles and responsibilities of the PTAs on the hospital ward. Although some nurses were already convinced of the added value that PTAs could bring to tasks such as patient admission and discharge, they uniformly expressed that such an expansion of the role could only be considered viable and effective when there are PTAs assigned to the hospital ward. This continuity, they believed, is crucial in establishing a deeper understanding of the ward's dynamics and developing a level of trust and knowledge necessary for additional responsibilities.

Nurse 4: “I think it would be a whole different story if you have one or two PTAs permanently employed on your hospital ward. When you look at the whole concept of admission, hospitalization and discharge, you look at a competency profile of what a PTA is on our ward.”

### 3.3. Patient Safety and Quality of Care

#### 3.3.1. Nurses' Time Saving and Decreased Workload

Nurses expressed a consistent perspective that the implementation of PTAs not only has resulted in a saving of nurses' time but has also given them the opportunity to redirect their focus to other critical aspects of patient care and contribute to the overall quality of care provided. All participants mentioned a reduced nursing workload on pharmaceutical care. They indicated that this shift in responsibilities enabled them to make more patient rounds, allocate more time for the admission of new patients, and dedicate more attention to individual direct patient care. In addition, it provided the flexibility to engage in meaningful conversations with patients who may benefit from such interactions, addressing their emotional and psychosocial needs.

Nurse 4: “For our night nurses, it's a return of, I think, two thirds of the medication preparation time.”

Nurse 11: “I think that's a good thing. Because during the night… For example, last week I had two seriously ill people on my ward. I had the opportunity to check on them every two hours, which I couldn't do before.”

Nurse 16: “It's just a heavy weight that has been lifted from your shoulders during the night… you also have more time for the patients as well.”

#### 3.3.2. Lower Risk of Medication Errors

The participants described the challenges faced by night nurses, who are frequently disturbed by patient calls, medication rounds, and overnight admissions. These factors, in combination with natural daily rhythm, are seen to contribute to a greater risk of reduced alertness during night shift hours. Recognizing potential risks, particularly related to the dispensation of medication and its higher risk of medication errors, participants believed that PTAs decrease the risk of errors because they are dedicated to the singular task of medication dispensing during their time on the hospital ward.

Nurse 3: “They (PTAs) can be specifically working on that, they work on their medication for two hours continuously. The night nurse has never been able to do that, meanwhile there are patient calls, someone doesn't get well… They (PTAs) are doing that, they are not being disturbed by anyone, so you will certainly have fewer mistakes than you are constantly distracted.”

Nurse 4: “I think, according to the time of night, the workload, the whole patient care you are doing, you are not always alert to prepare medication during the night. A PTA is to focus on one task, which is always an added value.”

#### 3.3.3. Creating a Relationship of Trust and Developing Competencies

As mentioned in the previous theme, all participants considered it important to consistently assign the same hospital wards to the same PTAs. This approach has several advantages, offering PTAs an in-depth understanding of prevalent pathologies, improving clinical reasoning skills, and fostering team integration. Some head nurses proposed the idea of permanently stationing one or two PTAs on the hospital ward; in their belief, this could bring additional value to the nursing team by cultivating even stronger relationships, efficient communication, and ensuring a consistently high level of expertise in medication management.

Nurse 13: “It would be more ideal, I think, if we had someone on the ward who was a member of the team and was actually fully in charge of the medication from preparation to administration of the medication.”

Nurse 14: “I really hope they are going to be permanent people who work there, what would make sense. Because you know each other in the long run. You can tune into each other and you know what to expect from each other. If it's someone else every time, you don't know either.”

## 4. Discussion

In exploring nurses' views on the implementation of PTAs for medication dispensation on hospital wards, three main themes have emerged: “organization of medication management,” “patient safety and quality of care,” and “role development and collaboration.” PTAs are valuable resources to provide safe, efficient, and high-quality pharmaceutical care.

In the theme *“organization of medication management*,” facilitators and barriers were identified. Clear protocols, comprehensive training, PTA supervision, and efficient task allocation streamlined the process, reducing nurse workload, and improving efficiency. Regarding continuity in assigning PTAs, the disparities in perspectives reflect the dynamic and context-specific nature of healthcare settings. Optimal scheduling and PTA allocation depend on factors, such as patient awareness, ward specialization, and demand for nursing care at different times. On the contrary, the barriers included inadequate PTA numbers and possible medication errors due to a lack of standardized procedures. To enhance this aspect, healthcare organizations should consider investing in resources and establishing standardized procedures. This might involve ensuring an adequate number of PTAs and implementing clear guidelines for their role. By doing so, organizations can optimize the integration of PTAs into nursing practices, thus improving overall medication management [26].

The second theme, “*role development and collaboration*,” emphasized a unified healthcare team. Facilitators, such as clear scopes of practice for PTAs, collaborative work environments, and mutual respect between PTAs and nurses, improve collaboration and task transition [27]. However, barriers such as role ambiguity, inadequate nurse-PTA communication, and resistance to new responsibilities can impede this collaboration.

The third theme, “*patient safety and quality of care*,” highlighted the advantages of implementing PTAs in maintaining high standards of patient safety. Kjeldsen et al. [15] reported that the dispensation of medication by PTAs on a geriatric ward resulted in reducing interruptions, dispensing time, and reported medication errors. Miscommunication between nurses and PTAs could pose a threat to high-quality medication management. Therefore, healthcare teams must prioritize effective communication, double-check procedures, and continuous training to improve patient safety and quality of care [28, 29].

Implementing PTAs on hospital wards presents a multifaceted opportunity for nursing management. While the practical benefits are undeniable, ensuring successful integration requires a comprehensive approach. Beyond facilitating collaboration and fostering communication, nursing managers must navigate ethical considerations and provide adequate training for PTAs. Addressing potential challenges like role ambiguity and proactively managing interprofessional relationships are also crucial [30]. Continuously evaluating the impact of PTAs on patient outcomes and healthcare delivery efficiency allows for optimizing their contribution. By embracing this broader perspective, nursing management can unlock the full potential of PTAs, ultimately enhancing patient care, staff satisfaction, and the overall healthcare delivery system [31, 32].

To prevent missed nursing care, such as patient self-management support, education, psychological support, and shared decision-making, and to promote interprofessional collaboration, team members should have a clear understanding of each role. Creating a cohesive collaboration among nurses, physicians, and pharmacists is crucial to improve the quality of pharmaceutical care and ultimately improve patient outcomes. A precise description of the role of pharmaceutical care is necessary. The NuPHAC-EU framework was developed for this purpose, describing the role of nurses in pharmaceutical care, encompassing patient, and professional networks in seven domains with 26 tasks. Nurses can carry out tasks with varying degrees of autonomy based on contextual factors. This framework provides a structured approach and facilitates discussions among healthcare professionals about shared responsibilities and tasks. Adopting this framework within healthcare organizations can help redefine the roles and responsibilities of nurses and PTAs. It encourages transparent communication among a variety of healthcare professionals and provides essential support during role transitions through comprehensive education and training initiatives [28].

Previous studies on the collaboration between the PTA and nurses suggest a lower risk of medication errors and improved quality of care [12–15]. The perspectives of nurses in this study support the idea that this collaboration is beneficial in healthcare settings. However, to fully optimize PTAs implementation on hospital wards, it is imperative to investigate the experiences and perceptions of these PTAs. Understanding their perspectives, challenges, and insights can provide valuable information to refine the integration process, address potential barriers, and maximize the positive impact of PTAs in medication management within healthcare settings.

This study did not directly measure effects, but the existing literature suggests potential benefits of implementing PTAs, including cost savings, increased nursing time for (in)direct patient care activities, and potentially improving workflow efficiency [12–15, 29, 33]. To understand the PTAs' impact, future research exploring measurable effects, cost implications, and broader outcomes could provide additional information on their effectiveness and contribution to healthcare settings.

Although interviews were conducted across various hospital wards, representing different specialities such as internal medicine, surgery, and geriatrics, focusing on the implementation of PTAs within a single healthcare organization limits the generalizability of the findings to other healthcare settings. However, by exploring the experiences and perspectives of nurses within this specific context, this study offers valuable insights that can contribute to theoretical generalization with caution. These findings may not be directly applicable to all healthcare settings, particularly those with different cultural contexts, organisational structures, policies, or practices. Nevertheless, the identified themes can provide valuable inspiration and transferable knowledge for other healthcare organizations considering the implementation of PTAs on their hospital wards. This limitation emphasizes the need for further research that encompasses multiple healthcare organizations or settings to provide a more comprehensive and diverse understanding of the implementation and perceptions surrounding PTAs in medication management.

To ensure the robustness of our data analysis and findings, a variety of rigorous methods and techniques were used. Triangulation involved a meticulous cross-validation process. This regular cross-check meetings between the two researchers allowed for critical and sense-making discussion of data interpretations. In addition, discussions within the project group provided a valuable collaborative perspective that served to validate the findings and mitigate potential bias. Furthermore, the thematic presentation and discussion of the themes with the participants were important to maintain the authenticity of our interpretations. This interactive dialogue provided a deeper exploration of the participants' experiences and insights, reducing the risk of superficial or false interpretations, and ensured that the data analysis was embedded in the participants' experiences.

## 5. Conclusion

This study provided a comprehensive understanding of nurses' perspectives on the implementation and advancement of the role of PTAs in the dispensation of medications on the hospital ward. The recognition of PTAs by nurses as valuable contributors in medication management highlights their potential to positively impact healthcare settings. Understanding and embracing this perspective could facilitate further integration and optimization of PTAs within hospital wards, benefiting both healthcare professionals and, most importantly, the patients they serve.

## Figures and Tables

**Table 1:** Participants' characteristics.

Characteristics of nurses (*n* = 16)	*n* (%)/Mean (range)
Gender	
Male	5 (31)
Female	11 (69)
Age, mean (range), y	40.8 (24–58)
Educational level	
EQF and ISCED-P^*∗*^ level 5	11 (69)
EQF and ISCED-P^*∗*^ level 6	5 (31)
Work experience	
6 months -<5 y	3 (19)
5–10 y	2 (12)
>10 y	11 (69)

^*∗*^EQF: European Qualification Framework and ISCED: International Standard Classification of Education 2011.

## Data Availability

The data used to support the findings of this study are available from the corresponding author upon reasonable request.
